# Th17 cells inhibit CD8^+^ T cell migration by systematically downregulating CXCR3 expression via IL-17A/STAT3 in advanced-stage colorectal cancer patients

**DOI:** 10.1186/s13045-020-00897-z

**Published:** 2020-06-05

**Authors:** Dan Wang, Weina Yu, Jingyao Lian, Qian Wu, Shasha Liu, Li Yang, Feng Li, Lan Huang, Xinfeng Chen, Zhen Zhang, Aitian Li, Jinbo Liu, Zhenqiang Sun, Junxia Wang, Weitang Yuan, Yi Zhang

**Affiliations:** 1 Biotherapy Center, The First Affiliated Hospital of Zhengzhou University, Zhengzhou, 450052 Henan People’s Republic of China; 2 Cancer Center, The First Affiliated Hospital of Zhengzhou University, Zhengzhou, 450052 Henan People’s Republic of China; 3 Department of Anorectal Surgery, The First Affiliated Hospital of Zhengzhou University, Zhengzhou, 450052 Henan People’s Republic of China; 4 Department of Gastrointestinal Surgery, The First Affiliated Hospital of Zhengzhou University, Zhengzhou, 450052 Henan People’s Republic of China; 5 School of Life Sciences, Zhengzhou University, Zhengzhou, 450052 Henan People’s Republic of China; 6 Henan Key Laboratory for Tumor Immunology and Biotherapy, Zhengzhou, 450052 Henan People’s Republic of China

**Keywords:** Colorectal cancer, CD8, CXCR3, IL-17A, Th17 cells

## Abstract

**Background:**

CD8^+^ T cell trafficking to the tumor site is essential for effective colorectal cancer (CRC) immunotherapy. However, the mechanism underlying CD8^+^ T cell infiltration in colorectal tumor tissues is not fully understood. In the present study, we investigated CD8^+^ T cell infiltration in CRC tissues and the role of chemokine–chemokine receptor signaling in regulation of T cell recruitment.

**Methods:**

We screened chemokines and cytokines in healthy donor and CRC tissues from early- and advanced-stage patients using multiplex assays and PCR screening. We also utilized transcription factor activation profiling arrays and established a xenograft mouse model.

**Results:**

Compared with tumor tissues of early-stage CRC patients, CD8^+^ T cell density was lower in advanced-stage tumor tissues. PCR screening showed that CXCL10 levels were significantly increased in advanced-stage tumor tissues. CXCR3 (the receptor of CXCL10) expression on CD8^+^ T cells was lower in the peripheral blood of advanced-stage patients. The migratory ability of CD8^+^ T cells to CXCL10 depended on CXCR3 expression. Multiplex arrays showed that IL-17A was increased in advanced-stage patient sera, which markedly downregulated CXCR3 expression via activating STAT3 signaling and reduced CD8^+^ T cell migration. Similar results were found after CD8^+^ T cells were treated with Th17 cell supernatant. Adding anti-IL-17A or the STAT3 inhibitor, Stattic, rescued these effects in vitro and in vivo. Moreover, survival analysis showed that patients with low CD8 and CXCR3 expression and high IL-17A levels had significantly worse prognosis.

**Conclusions:**

CD8^+^ T cell infiltration in advanced-stage tumor was systematically inhibited by Th17 cells via IL-17A/STAT3/CXCR3 axis. Our findings indicate that the T cell infiltration in the tumor microenvironment may be improved by inhibiting STAT3 signaling.

## Introduction

Colorectal cancer (CRC) is one of the most prevalent cancers worldwide and is the third leading cause of malignant tumor-related death. Adaptive intratumor immunity can contribute to tumor elimination or progression. Several preclinical and clinical studies have suggested that increased CD8^+^ T cell infiltration is correlated with a better prognosis for CRC patients [1–4]. Decreased CD8^+^ T cell density has been shown in advanced-stage CRC patients and those with nodal metastasis [5]. Moreover, patients with hot tumors, displaying a high degree of T cell infiltration, exhibit better responses to immune checkpoint blockade [6, 7]. Therefore, tumor-infiltrating CD8^+^ T cells are considered the major effector immune cells in antitumor immunity [8, 9]. However, the mechanisms underlying CD8^+^ T cell infiltration in tumor remains unclear.

Chemokine–chemokine receptor signaling is one of the major factors regulating T cell recruitment to tumors and antitumor immunity [10–12]. The CXCR3 receptor and its cognate ligands, CXCL9, CXCL10, and CXCL11, which mainly secreted from tumor cells, have been implicated in recruiting CD8^+^ T cells to stroma [13, 14]. Consistent with these studies, CD8^+^ T cell accumulation in the tumor microenvironment (TME) was found to depend on CXCR3 [15]. In addition, increased CXCL10 expression in the TME is associated with increased overall survival (OS) in CRC [16, 17]. On the other hand, several studies have reported that elevated CXCL10/CXCR3 levels were significantly associated with poor survival across all stages of CRC and are independent markers for predicting liver metastasis [18, 19]. Nevertheless, the clinical significance of CXCL10/CXCR3 during CRC progression and the regulatory mechanism for activating CXCL10/CXCR3 signaling remain unknown. Recent studies have shown that the expression of CXCR3 on tumor-specific CD8^+^ T cells is associated with a better survival of patients with advanced metastatic melanoma [20]. CXCR3-positive innate CD8^+^ T cells express high transcript levels of antiapoptotic genes and produce significantly increased levels of interferon (IFN)-γ and granzyme B [21, 22]. Thus, increased CXCR3 expression on CD8^+^ T cells is thought to be desirable for active immunotherapy.

The TME was shown to be immunosuppressive, which is a key reason why most cancer immune therapies often display limited clinical efficacy [23]. IL-17A is an inflammatory cytokine secreted by different cell types, including CD4^+^ T helper cells (Th17), CD8^+^ T cells, and γδT cells [24, 25]. Th17 cells can facilitate tumor growth by promoting angiogenesis and/or inhibiting immune responses via myeloid derived suppressor cells [26, 27]. IL-17A may also enhance tumor growth by suppressing CD4^+^ and CD8^+^ T cell infiltration [28, 29]. Recently, however, several studies have indicated that IL-17A exerts antitumor effects via the immune cells; in IL-17A^−/−^ mice, IFN-g^+^CD4^+^ T cells, IFN-g^+^CD8^+^ T cells, and IFN-g^+^ natural killer (NK) cells were found decreased in tumor-draining lymph nodes [30] and IL-17A stimulation of tumor cells resulted in migration of CD3^+^ T cells and NK cells [31]. The role of IL-17A on tumor progression has not yet been clearly determined; therefore, studying the effect of IL-17A on the infiltration of immune effector cells in patients with CRC is warranted.

In this study, we investigated the levels of CD8^+^ T cell infiltration in tumor tissues during CRC progression and the role of chemokine–chemokine receptor signaling involving in the regulation of T cell recruitment. Our results showed that the accumulation of CD8^+^ T cells in advanced-stage tumor was systematically inhibited by Th17 cells via IL-17A/STAT3/CXCR3 axis. These findings identify a potential therapeutic target for turning cold tumors into hot tumors, which can improve immunotherapy efficacy.

## Materials and methods

### Clinical sample selection

A total of 125 specimens (tumor and peripheral blood (PB) samples) were freshly obtained from the Department of Anorectal Surgery of the First Affiliated Hospital of Zhengzhou University (Zhengzhou, China). Healthy donors (HD, *n* = 50) were enrolled from the same hospital’s physical examinations center. Paraffin-embedded tissue samples from CRC patients (*n* = 75) diagnosed between 2011 and 2013 were obtained from the Pathology Department. All patients did not receive any therapeutic intervention such as chemo- or radiotherapy. All CRC patients were diagnosed histologically. Age- and sex-matched controls were selected and patients were staged according to the UICC-TNM classification. Early-stage patients included patients with stage I and II. Advanced-stage patients included patients with stages III and IV. The clinical data of the patients are shown in Table 1. Samples used in this study were approved by the Ethics Committee of the First Hospital of Zhengzhou University (approval number: Science-2010-LW-1213) and informed consent was obtained from each patient with available follow-up information.

**Table 1:** Characteristics of patients with colorectal carcinoma

Characteristics	CRC patients (*n* = 125)	
	Number	%
Gender		
Male	53	42.4
Female	72	57.6
Age (years)		
< 60	59	47.2
≥ 60	66	52.8
Location		
Colon	56	44.8
Rectum	69	55.2
Tumor size		
< 40 mm	49	57.5
≥ 40 mm	76	42.5
Pathological type		
Adenocarcinoma	105	84.0
Others	20	16.0
Lymph node metastasis		
Yes	76	60.8
No	49	39.2
Distant metastasis		
Yes	36	28.8
No	89	71.2
TNM Stage		
I	10	8.0
II	39	31.2
III	40	32.0
IV	36	28.8
Differentiation		
Low	10	8.0
Low moderate	30	24.0
Moderate	85	68.0

### Flow cytometry analysis

Mononuclear cells from tumors and PB were isolated and stained for 30 min at 4 °C using saturating concentrations of the following antibodies: anti-human CD3, CD8, CD4, CD56, CXCR3, and 7-AAD antibodies and anti-mouse CD3, CD8, and CXCR3 antibodies (BioLegend, San Diego, CA). Intracellular staining of granzyme B, perforin, IFN-γ, and IL-10 was performed as previously described [32]. Cells were then analyzed by flow cytometry (BD FACSCanto II, BD Biosciences, Franklin Lakes, NJ).

### Immunohistochemistry and immunofluorescence

Specimens from CRC patients and mice were formalin fixed, sectioned, and embedded into paraffin for immunohistochemistry. Immunofluorescence was performed with frozen samples and freshly fixed cells. The following antibodies were used: anti-CD8 (1:500), anti-CXCL10 (1:300), anti-CD4 (1:300), anti-IL-17A (1:300), and anti-CXCR3 (1:300; all Abcam, Cambridge, UK). Immunohistochemistry and immunofluorescence were performed as described elsewhere [33, 34]. For immunohistochemistry, three fields of view per sample were imaged. The intensity of immunostaining was considered when analyzing the data. The percentage scoring of immunoreactive cells was as follows: 0 (< 10%), 1 (10–40%), 2 (40–70%), and 3 (> 70%). Staining intensity was visually scored and stratified as follows: 0 (negative), 1 (yellowish), 2 (light brown), and 3 (dark brown). Immunoreactivity scores (IRS) were obtained by multiplying the two items to a total score and ranged from 0 to 9. Protein expression levels were further analyzed by classifying IRS values as low (based on an IRS value ≤ 5) and as high (based on an IRS value > 5).

### Transwell assays

Isolation of CD8^+^ T cells from HD samples was performed with CD8 magnetic beads (Miltenyi Biotech, Bergisch Gladbach, Germany) according to manufacturer’s instructions. Freshly isolated CD8^+^ T cells, anti-CXCR3 (10 ng/mL, Abcam), or Stattic (10 nM; S7024; Selleck Chem, Houston, TX)-treated CD8^+^ T cells as well as peripheral blood mononuclear cells (PBMCs) from HDs were seeded on the upper chambers of a 5.0-μm pore Transwell. Supernatants of tumor tissue, anti-CXCL10 (100 ng/mL, Abcam), or recombinant human (rh) CXCL10 (Peprotech, Rocky Hill, NJ) were added to the lower chambers. CD8^+^ T cells in the upper chambers were pretreated with rhIL-17A (20 ng/mL), Stattic (10 nM), and/or the supernatants of enriched Th17 cells (5 × 10^6^) with or without anti-IL-17A (10 ng/mL) for 48 h in vitro. Migrated cells in the lower chambers were then counted and analyzed by flow cytometry after 12 h.

### Western blotting

Total protein was extracted after lysing cells in RIPA lysis buffer supplemented with protease inhibitor cocktail (P8340; Sigma-Aldrich, St. Louis, MO) and phosphatase inhibitor cocktail 2 (P5726, Sigma-Aldrich) as previously described [35]. The following primary antibodies were used: rabbit anti-phosphor-STAT3 (1:1000; Cell Signaling Technology, Danvers, MA), mouse anti-CXCR3 (1:1000, Cell Signaling Technology), and mouse anti-b-actin as control (1:3000, Cell Signaling Technology). Primary antibodies were detected with goat polyclonal rabbit or mouse IgG antibodies (1:1000, Cell Signaling Technology).

### Enzyme linked immunosorbent assay (ELISA)

IL-17A concentrations in HD and CRC patient sera were measured using ELISA (R&D Systems Inc., Minneapolis, MN) according to manufacturer’s instructions.

### Quantitative reverse-transcription PCR (qRT-PCR)

Total RNA was extracted using TRIzol reagent (Invitrogen, Carlsbad, CA), after which cDNA was reverse transcribed following manufacturer’s instructions. qRT-PCR was then performed using SYBR Green, and GAPDH was used as an internal control. Relative expression levels were determined using the 2^−ΔΔCt^ method. Primers used are listed in Supplementary Table 1.

### Multiplex assays

Cytokine levels in the sera of CRC patients and supernatants from tumor tissues and paired normal tissues were analyzed using a Multi-Analyte Flow Assay Kit (BioLegend) that includes 13 human cytokines according to manufacturer’s instructions.

### Transcription factor activation profiling array

CD8^+^ T cells sorted from PBMCs of HDs were treated with 20 ng/mL rhIL-17A. Nuclear proteins were isolated with a Nuclear Extraction Kit (SK-0001; Signosis, Santa Clara, CA) and analyzed using a 96-well plate Transcription Factor (TF) Activation Profiling Array (FA1002; Signosis) according to manufacturer’s instructions. Quantification of each transcriptional factor was normalized as the fold change of TFIID activity.

### Identification of upstream signaling pathways

CXCR3 upstream signaling was screened using radar tools of the Gene-Cloud of Biotechnology Information (GCBI) database (www.gcbi.com.cn).

### Isolation of lymphocytes

Human CD8 magnetic beads (Miltenyi Biotec) were used for isolating CD8^+^ T cells from PBMCs according to manufacturer’s instructions. CXCR3^+^CD8^+^ cells and CXCR3^-^CD8^+^ cells were sorted from PBMCs using the MoFlo XDP cytometer (Beckman Coulter, Brea, CA). The positive rate of cells after purification was more than 90%.

### Enrichment of Th17 cells

Th17 cell culture and enrichment were carried out as described previously [36]. In brief, CD4^+^ memory T cells were isolated from freshly obtained PBMC using Memory CD4^+^ T Cell Isolation Kit (Miltenyi Biotech). The CD4^+^ memory T cells were treated with anti-CD3/CD28/CD2 beads for 7 d in the presence of recombinant IL-6 (40 ng/mL), IL-1b (10 ng/mL), IL-23 (50 ng/mL), neutralizing anti-IL-4 (0.5 mg/mL), and anti-IFN-g (5 mg/mL). Then cultured Th17 cells were enriched using an IL-17 Secretion Assay-Cell Enrichment and Detection Kit (130-094-542, Miltenyi Biotech). Cultured Th17 cells were further stimulated with cytostim (20 μL/mL) for 6 h at 37 °C. Then, the cells were labeled with IL-17 catch reagent, followed by incubation in closed tubes for 45 min at 37 °C under slow continuous rotation. The cells were then labeled with IL-17 detection antibody (PE) and anti-PE microbeads, followed by cell collection using magnetic separation with MS columns according to manufacturer’s instructions.

### Mouse studies

To generate a xenograft mouse model, 6-week-old female BALB/c nude mice were purchased from Vital River Laboratory Animal Technology Co. Ltd (Beijing, China) and randomly divided into groups. Luc-GFP-HCT116 cells (3 × 10^6^) were injected subcutaneously. 1 × 10^6^ CD8^+^ T cells were co-cultured with rhIL-17A (20 ng/mL) and/or Stattic (10 nM) for 48 h in vitro. Seven days after tumor cell implantation, pretreated CD8^+^ T cells were injected intravenously. After 48 h, CD8^+^ T cell infiltration in tumor tissues was examined via immunohistochemistry. To further evaluate the tumor growth, on day 0, CD8^+^ T cells, Th17 cells, or CD8^+^ T cells plus Th17 cells were injected intravenously. Next, 3.75 mg/kg Stattic was administrated to mice intraperitoneally every day for 1 week [37]. Tumorigenicity was evaluated using an in vivo imaging system (IVIS Lumina Series III; PerkinElmer, Waltham, MA) every 6 days. Mice were then sacrificed, after which tumors were harvested and blood collected from the tail vein for further analysis. All animal studies were approved by the Institutional Animal Care and Use committee of the First Affiliated Hospital of Zhengzhou University.

### Statistical analysis

Data are expressed as the mean ± standard deviation. Differences among groups were compared using Student’s *t* test, chi-square test, and one-way ANOVA. OS curves were plotted according to the Kaplan-Meier method. Correlation between two variables was analyzed by Spearman’s rank-order correlation. Statistical analyses were performed using the GraphPad Prism 5 software (GraphPad Software Inc., La Jolla, CA). *P* values < 0.05 were considered statistically significant.

## Results

### Early- and advanced-stage CRC patients exhibit unbalanced expression levels of CD8 and CXCL10

**Fig 1:**
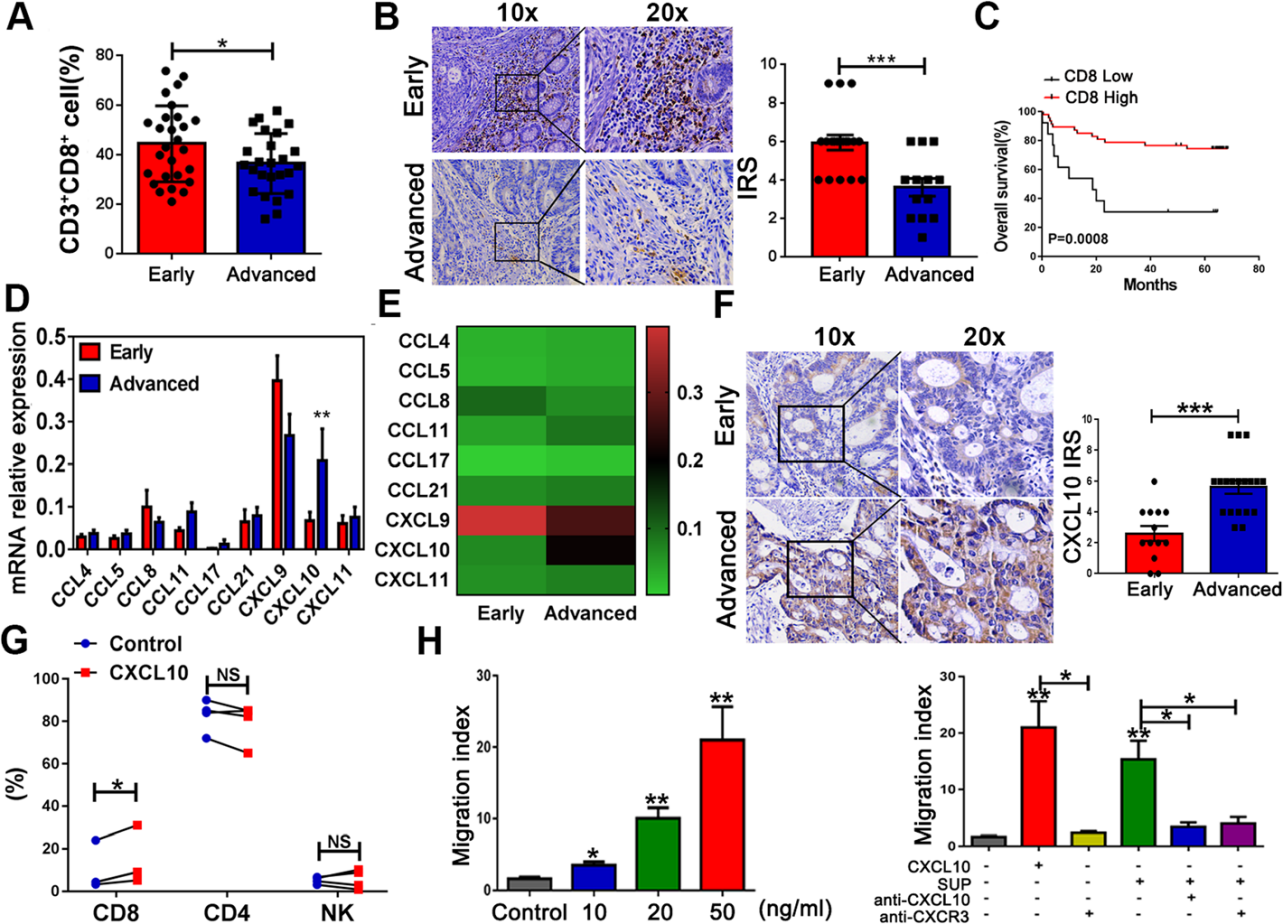
Decreased infiltration of CD8^+^ T cells and increased CXCL10 secretion in tumor tissues from advanced-stage CRC patients. **a** FACS analysis of CD8^+^ T cell infiltration in tumor tissues of early- and advanced-stage CRC patients. **b** CD8 expression was analyzed by immunohistochemistry in tumor tissues; magnification, × 200 (left). Right panel, IRS (0–9) = intensity score (0–3) × percentage score (0–3). **c** Kaplan-Meier OS curves of CRC patients, presented as high-CD8 or low-CD8 expression groups based on the log-rank statistic test (*n* = 70). High, IRS > 4; low, IRS ≤ 4. **d**, **e** Relative expression levels of effective immune cell-associated chemokines in CRC tumor tissues determined by real-time PCR. f CXCL10 expression was analyzed by immunohistochemistry. **g** Conditioned media were cultured in the lower chambers of a Transwell plate with or without rhCXCL10. Migrated PBMCs from HDs were collected and assessed via flow cytometry. Each line represented a different HD. **h** The migratory ability of purified CD8^+^ T lymphocytes (purity > 90%) from HDs to rhCXCL10 at different concentrations (left). Supernatants of primary tumor tissues were added alone or with CXCL10 (50 ng/mL)-specific neutralizing antibodies as indicated. After incubation, CD8^+^ T cells alone or cells pretreated with anti-CXCR3 that migrated into the lower chambers were collected and counted. The migration index was calculated by dividing the number of cells that migrated in the indicated groups by the number that migrated in the control groups (right). *P < 0.05, **P < 0.001, ***P < 0.0001; NS non-significant

As tumor-infiltrating CD8^+^ T cells are indicators of an active host immune response against cancer [4], we quantified the infiltrating CD8^+^ T cells in tumor tissues of early- and advanced-stage CRC patients. CD8^+^ T cell density was found lower in advanced-stage tumor tissues compared with early-stage tumor tissues, and high expression of CD8 was associated with a favorable prognosis (Fig. 1a–c). Given that T cell infiltration of tumors is a multi-step process that is mediated, in part, by chemokine-chemokine receptor pathways [38], we examined the potential chemokines contributing to T cell infiltration and found that CXCL10 expression were significantly increased in advanced-stage tumor tissue compared with early-stage tumor tissues. Other chemokines exhibited no significant difference in their expression levels, which were also inconsistent with CD8^+^ T cell infiltration patterns (Fig. 1d, e). These findings were similar at the protein level by IHC (Fig. 1f). The staining results showed that CXCL10 was predominately produced from tumor cells rather than from stroma cells (Fig. 1f). We then utilized Transwell migration assays to test the role of CXCL10 in cell recruitment and found that CD8^+^ T cell migration increased significantly after CXCL10 treatment; in contrast, CD4^+^ T cells and NK cell migration remained unchanged (Fig. 1g). Next, CD8^+^ T cells were isolated from freshly obtained HD PBMCs using magnetically activated cell sorting (MASC) and the purity obtained was greater than 90% (data not shown). CD8^+^ T cell movement was also markedly enhanced along with increasing concentrations of rhCXCL10 (Fig. 1h). We further investigated the regulatory effects of CXCL10/CXCR3 on CD8^+^ T cell migration using supernatants from freshly obtained CRC tumor tissues. Supernatants derived from tumor tissues markedly facilitated CD8^+^ T cell migration, which was attenuated by anti-CXCL10 or anti-CXCR3 neutralizing antibodies. These findings indicate that CXCL10 attracts CD8^+^ T cell migration. This attraction was dependent on CXCR3, as a decreasing ratio of CXCR3^+^CD8^+^ cells to CXCR3^-^CD8^+^ cells led to a significantly reduced chemotactic ability of CD8^+^ T cells in the presence of the same concentrations of rhCXCL10. When the ratio decreased to 1:8, increased concentrations of rhCXCL10 also did not affect the chemotactic ability of CD8^+^ T cells (Supplementary Figure 1). Thus, we theorized that CXCR3 expression on CD8^+^ T cells plays an important role in the degree of CD8^+^ T cell infiltration in tumor tissues of CRC patients.

### Advanced CRC blood exhibit low expression levels of CXCR3 on CD8^+^ T cells

**Fig. 2:**
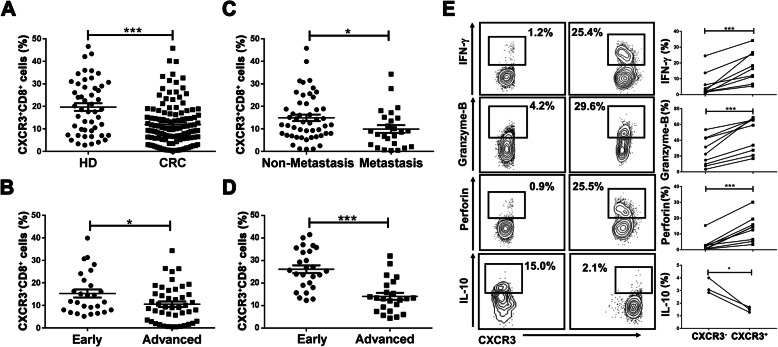
Decreased CXCR3 expression on CD8^+^ T cells in the PB of advanced-stage CRC patients. **b** PBMCs were isolated from the PB of CRC patients (*n* = 125) and HDs (*n* = 50) and analyzed by flow cytometry. Representative dot plots are shown. Data are presented as the percentage of CXCR3^+^CD8^+^ T cells in total CD8^+^ T cells. **b**, **c** The percentage of CXCR3^+^CD8^+^ T cells in the PBMCs of early-stage, advanced-stage, non-metastasis, and metastasis CRC patients. **d** The percentage of CXCR3^+^CD8^+^ T cells in tumor tissues of early-stage and advanced-stage patients. **e** The expression of IFN-γ-, granzyme-B-, perforin-, and IL-10-producing CD8^+^ T cells in total CD8^+^ T cells from PBMCs of CRC patients. The results for gating cells on the CXCR3 negative population are shown in the left panels and for gating on the CXCR3 positive population on the right. Each line represented a different patient.*P < 0.05, ***P < 0.0001

Previous studies have shown that CXCR3, the only receptor for CXCL10, facilitates CD8^+^ T cell recruitment in inflammatory and malignant diseases [36, 39]; thus, we examined CXCR3 expression on CD8^+^ T cells in PB using flow cytometry. Compared with CD8^+^ T cells from HDs, CXCR3 expression was found decreased in CRC patients (Fig. 2a). Interestingly, the percentage of CXCR3^+^CD8^+^ T cells was also lower in advanced-stage CRC compared with early stage, which was consistent with CD8^+^ T cell levels (Fig. 2b); a similar result was observed in CRC patients with distant metastasis compared with those without (Fig. 2c). CXCR3 expression levels were also found lower in tumor tissues with advanced-stage compared with early-stage CRC (Fig. 2d). To assess the function of CXCR3^+^CD8^+^ T cells, we identified the intracellular factors and found that the percentages of IFN-g granzyme B, and perforin were significantly higher and IL-10 levels were decreased in the CXCR3^+^CD8^+^ T cell subset compared with the CXCR3^-^CD8^+^ T cell subset (Fig. 2e). Taken together, these findings exhibited that the expression level of CXCR3 on CD8^+^ T cells was decreased in advanced-stage CRC PB.

### IL-17A downregulates the expression of CXCR3 on CD8^+^T cells

**Fig. 3:**
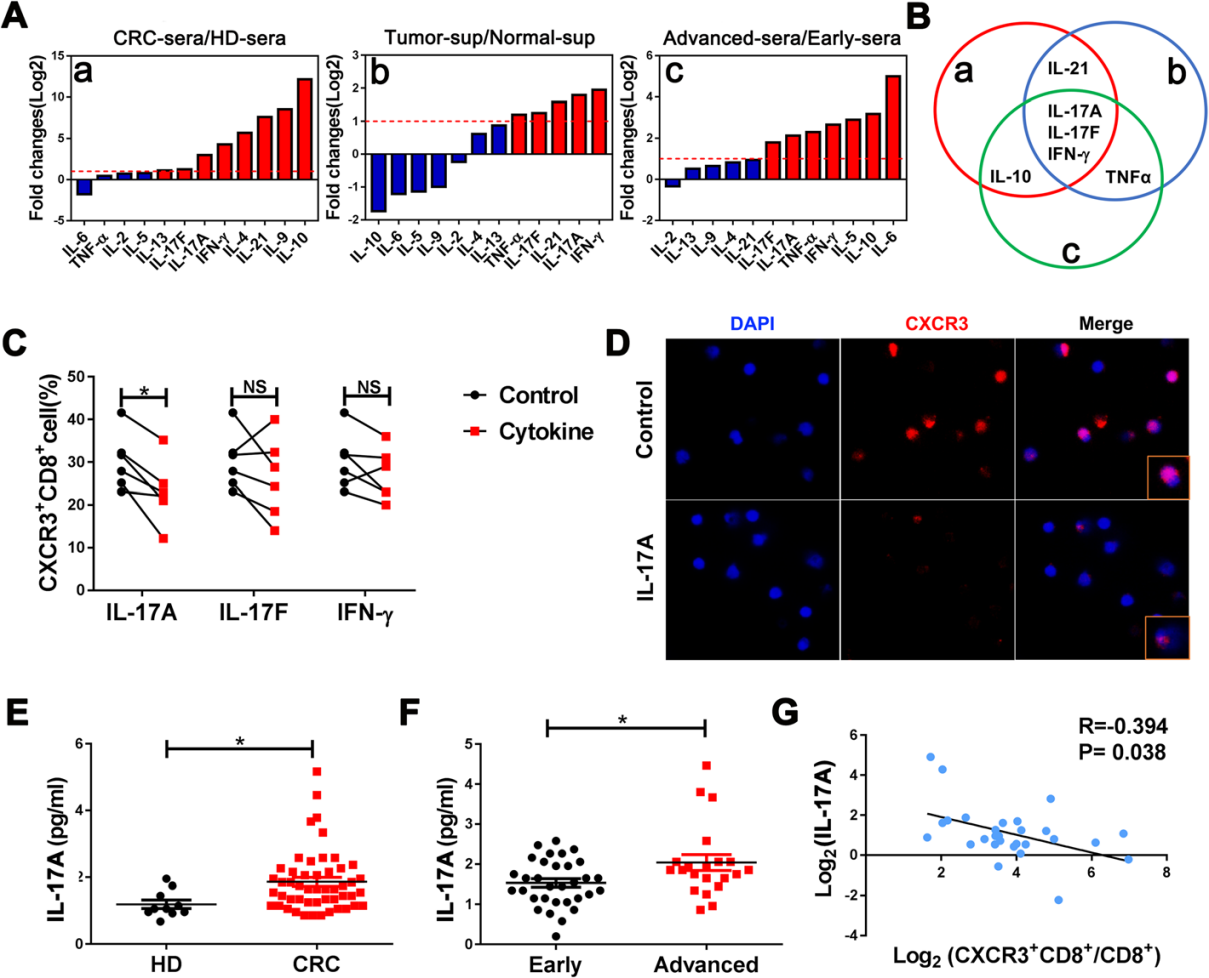
IL-17A downregulates CXCR3 expression on CD8^+^ T cells. **a** The expression of 13 cytokines in the sera of CRC patients (CRC-sera) vs. HD (HD-sera), sera of advanced-stage (advanced-sera) vs. early-stage CRC patients (early-sera), and supernatants of tumor tissues (tumor-sup) vs. normal tissues (normal-sup) were analyzed by multiplex assays. Fold changes were calculated with concentrations normalized with log2. **b** The intersection of the screened cytokines (fold changes ≥ 2) from a (red circle), b (blue circle), and c (green circle) of Fig. 3a. **c** FACS analysis of CXCR3^+^CD8^+^ T cell expression in CD8^+^ T cells treated with recombinant human proteins (20 ng/mL IL-17A, 20 ng/mL IL-17F, or 10 ng/mL IFN-γ) for 48 h. Each line represented a different HD. **d** Purified CD8^+^ T cells treated with or without rhIL-17A were subjected to immunofluorescence for CXCR3 (red) and DAPI (blue). **e**, **f** Concentrations of IL-17A (pg/mL) in sera obtained from CRC patients and HDs were measured using ELISA. **g** Correlation between the concentration of IL-17A and the percentage of CXCR3^+^CD8^+^ T cells in CD8^+^ T cells. Data were analyzed by Spearman’s rank correlation. *P < 0.05, NS non-significant

CXCR3 expression was reported to be regulated by several inflammatory factors, including IL-10, IL-4, and IL-15 [40–42]. To identify which factor(s) participate in the downregulation of CXCR3 on CD8^+^ T cells during CRC malignancy, we tested 13 inflammatory cytokines using a multiplex array in the following three groups: sera of CRC patients vs. HDs, sera of advanced-stage patients vs. early-stage patients, and supernatants of tumor tissues vs. paired normal tissues (Fig. 3a). We identified IL-17A, IL-17F, and IFN-g as significantly upregulated cytokines in the former of all the three groups (Fig. 3b). To further test which factors play important roles in regulating CXCR3 expression, we evaluated CXCR3 expression levels on CD8^+^ T cells after treatment with three recombinant proteins of IL-17A, IL-17F, and IFN-g and found that IL-17A markedly downregulated CXCR3 expression, whereas IL-17F and IFN-g did not lead to marked downregulation (Fig. 3c). Therefore, we decided to focus on the effects of IL-17A on CXCR3 expression regulation. Via immunofluorescence, we found that the fluorescence intensity and positive rate of CXCR3 expression can be inhibited by IL-17A (Fig. 3d). The results of ELISA further verified that IL-17A expression in sera was higher of CRC patients than of HDs and it was also higher in advanced-stage patients than in early-stage patients (Fig. 3e, f). Moreover, correlation analysis showed that IL-17A expression levels were negatively associated with the percentage of the CXCR3^+^CD8^+^T cells in PB (Fig. 3g). To verify whether IL-17A influenced the expression of CXCR3 ligands, we stimulated HCT116 cells with rhIL-17A. The results showed that CXCR3 ligands (CXCL9,10,11) were not changed significantly (Supplementary Figure 2). These results indicate that IL-17A plays a major role in downregulating CXCR3 expression on CD8^+^ T cells in advanced-stage CRC.

### IL-17A downregulates the expression of CXCR3 on CD8^+^ T cells via STAT3 phosphorylation

**Fig. 4:**
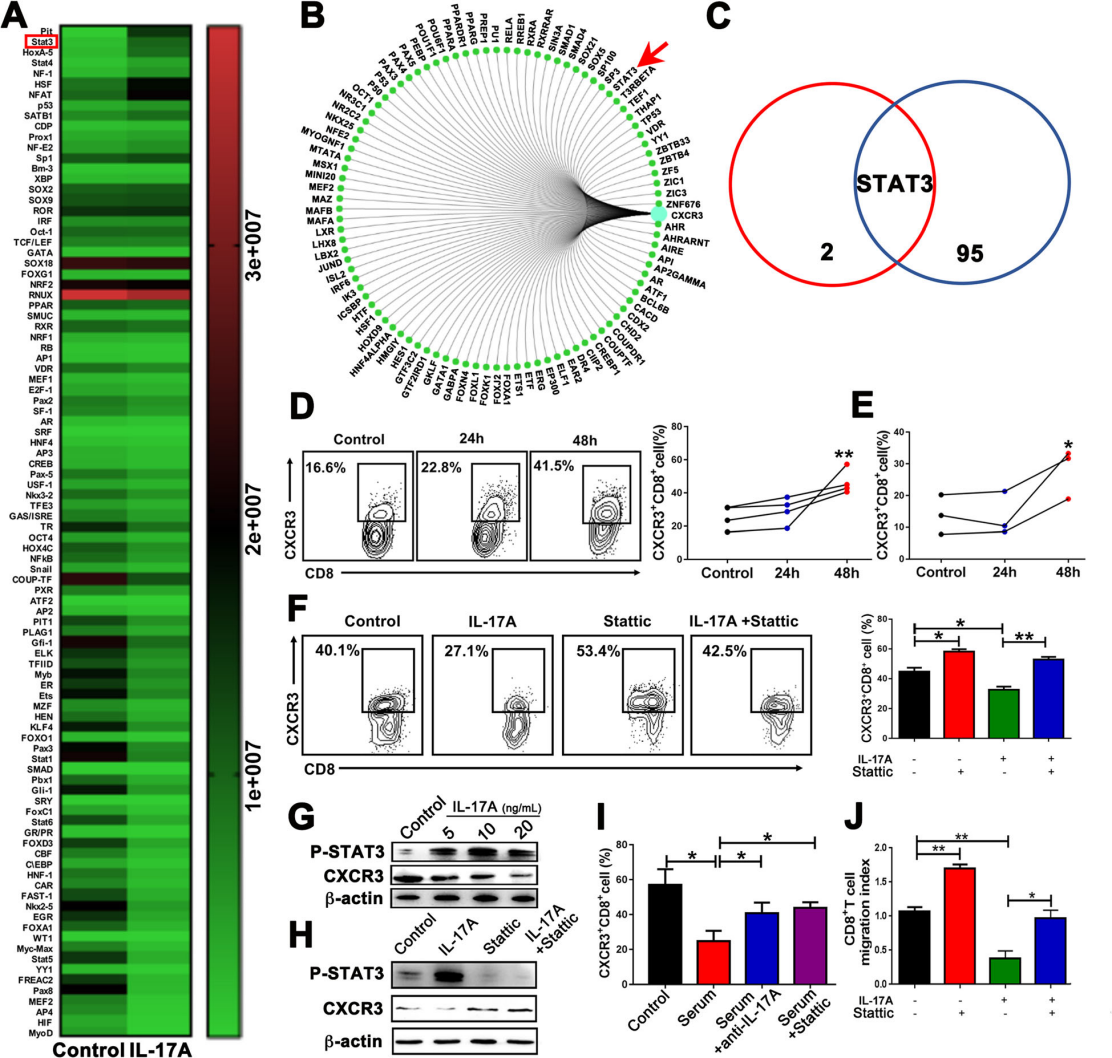
IL-17A inhibits CXCR3 expression on CD8^+^ T cells via STAT3 phosphorylation. **a** HD PB-derived CD8^+^ T cells were treated with rhIL-17A (20 ng/mL) for 48 h. Nuclei proteins were then subjected to multiplex profiling analysis for transcriptional factor activation. Activity was normalized with transcription factor IID and expressed as the relative light unit (RLU) of the transcriptional factor. **b** The upstream transcriptional factor of CXCR3 was identified via radar tools of the GCBI website. **c** The intersection of the screened transcriptional factors. Red circle: screened transcriptional factors (fold changes ≥ 4) from Fig. 4a; blue circle: upstream transcriptional factor of CXCR3 from Fig. 4b. **d**, **e** PBMCs from HDs (**d**) or purified CD8^+^ T cells (**e**) were treated with or without Stattic (10 nM). After 24 h and 48 h, CXCR3^+^CD8^+^ T cell expression was measured by flow cytometric analysis. Each line represented a different HD. **f** CXCR3^+^CD8^+^ T cell expression was measured after CD8^+^ T cells were treated with rhIL-17A (20 ng/mL) or Stattic (10 nM) for 48 h. **g** CD8^+^ T cells were incubated with rhIL-17A (5、10 and 20 ng/mL) for 48 h. P-STAT3 and CXCR3 expression levels were measured by western blotting. **h** CD8^+^ T cells were incubated with rhIL-17A (20 ng/mL) or Stattic (10 nM) for 48 h. **i**, **j** CXCR3^+^CD8^+^ T cell expression (**i**) or migratory ability of CD8^+^ T cells (**j**) was evaluated after treating sera collected from CRC patients (**i**) or RPMI-1640 conditional media (**j**) with rhIL-17A (20 ng/mL), anti-IL-17A (10 ng/mL), or Stattic. *P < 0.05, **P < 0.001

To explore the mechanisms underlying IL-17A regulation of CXCR3 expression on CD8^+^ T cells, we performed a multiplex screening assay for the DNA-binding activity of 96 transcriptional factors in IL-17A-treated CD8^+^ T cells sorted from the PBMCs of HDs. IL-17A treatment activated several transcriptional factors, including Pit, STAT3, and HoxA-5, as evidenced by a > 4-fold increase in their activity (Fig. 4a). To further examine which transcriptional factors are involved in regulating CXCR3, we used gene radar prediction from the GCBI database to search for upstream molecules of CXCR3, and 96 transcriptional factors were found (Fig. 4b). Based on the above results, STAT3 was identified as the key transcriptional factor of IL-17A regulating CXCR3 on CD8^+^ cells (Fig. 4c). To test this, we assessed CXCR3 levels on CD8^+^ T cells after treatment with Stattic, a STAT3 inhibitor. After incubating PBMCs with Stattic for 24 h and 48 h, the percentage of CXCR3^+^CD8^+^ T cells increased (Fig. 4d). Similar results were also obtained with purified CD8^+^ T cells sorted from PBMCs of HDs (Fig. 4e). Next, we evaluated whether inhibition of STAT3 signaling can reverse the IL-17A-induced inhibition of CXCR3 expression and found that this inhibitory effect was attenuated in CD8^+^ T cells treated with Stattic (Fig. 4f). In addition, western blot analysis showed that STAT3 phosphorylation increased, while CXCR3 levels decreased in a concentration-dependent manner after treatment with rhIL-17A (Fig. 4g). Consistent with previous results, the effect of rhIL-17A was markedly attenuated upon treatment with Stattic (Fig. 4h). Moreover, the percentage of P-STAT3^+^CD8^+^ T cells in total CD8^+^ T cells was found increased in PB of CRC patients vs. HDs and advanced-stage patients vs. early-stage patients (Supplementary Figure 3). Overall, our findings indicate that IL-17A inhibits CXCR3 expression through STAT3 signaling.

We further co-cultured sorted CD8^+^ T cells with sera collected from advanced-stage CRC patients and found that the percentage of CXCR3^+^CD8^+^ T cells was reduced; this percentage was upregulated after treatment with anti-IL-17A or Stattic (Fig. 4i). These results support the notion that CXCR3 expression on CD8^+^ T cells is inhibited by IL-17A and STAT3 signaling in the PB of advanced-stage CRC patients. Finally, Transwell migration assays demonstrated that IL-17A suppressed CD8^+^ T cell recruitment, which was rescued by the addition of Stattic (Fig. 4 j). Taken together, our findings indicate that STAT3 signaling is critical for the IL-17A-mediated regulation of CXCR3 expression.

### IL-17A mainly secreted from Th17 cells inhibits CD8^+^ T cell recruitment via STAT3

**Fig. 5:**
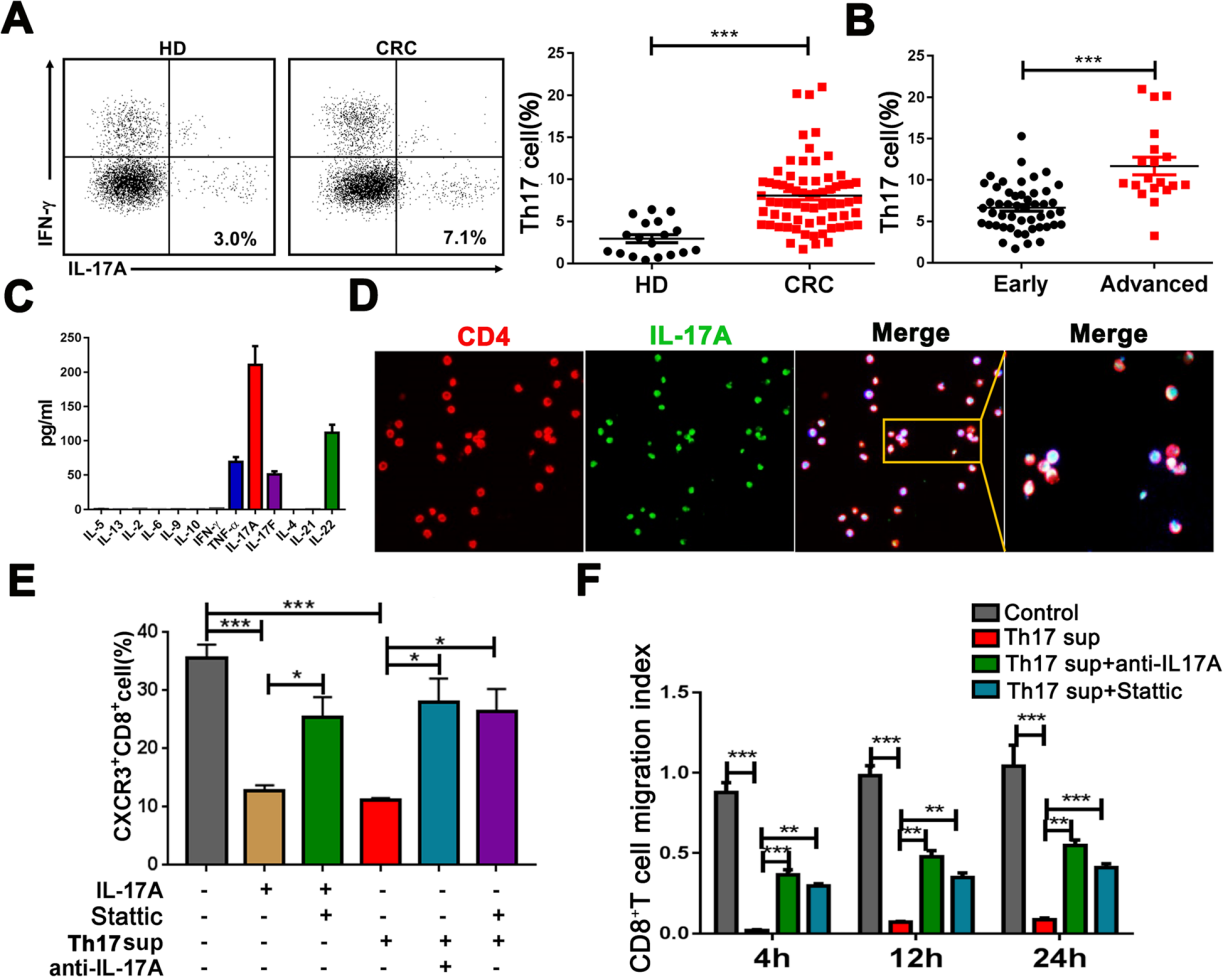
IL-17A, predominately secreted from Th17 cells, inhibits recruitment of CD8^+^ T cells via STAT3. **a**, **b** FACS analysis of Th17 cell (IFN-γ^-^IL-17^+^CD4^+^ T cells) expression in PB from CRC patients and HDs (**a**) as well as early- and advanced-stage CRC patients (**b**). **c** The expression of 13 cytokines in supernatants of Th17 cells induced by CD4^+^ T cells from HD PBMCs were analyzed by multiplex assays. **d** Enriched Th17 cells were subjected to immunofluorescence for IL-17A (green), CD4 (red), and DAPI (blue). **e**, **f** CXCR3^+^CD8^+^ T cell expression (**e**) or migratory ability of CD8^+^ T cell (**f**) after treatment with rhIL-17A (20 ng/mL), Stattic (10 nM), and/or the supernatants of enriched Th17 cells (5 × 10^6^) with or without anti-IL-17A (10 ng/mL) for 48 h. *P < 0.05, **P < 0.001, ***P < 0.0001

We performed immunofluorescence assays to investigate which cells secrete IL-17A and found that IL-17A was predominately produced from CD4^+^ T cells rather than from CD8^+^ T cells (Supplementary Figure 4). Thus, we examined the expression of Th17 cells in PB and found increased levels in CRC patients compared with HDs (Fig. 5a) that were especially higher in advanced-stage than early-stage patients (Fig. 5b). We next induced and enriched Th17 cells in vitro and the purity was more than 70% (Supplementary Figure 5). Using multiplex arrays, we identified that IL-17A was the most secreted cytokine among the 13 inflammatory cytokines (Fig. 5c). Furthermore, most enriched CD4^+^ T cells were found co-localized with IL-17A via immunofluorescence (Fig. 5d). In addition, CD8^+^ T cells treated with conditional medium collected from Th17 cells exhibited a decreased percentage of CXCR3^+^CD8^+^ T cells, which was rescued after adding anti-IL-17A or Stattic (Fig. 5e). The same trend was found for the migratory ability of CD8^+^ T cells (Fig. 5f). These findings indicate that Th17 cells inhibit the recruitment of CD8^+^ T cells via IL-17A/STAT3 signaling.

### Stattic rescues the Th17-induced inhibition of CD8^+^ T cell recruitment in vivo

**Fig. 6:**
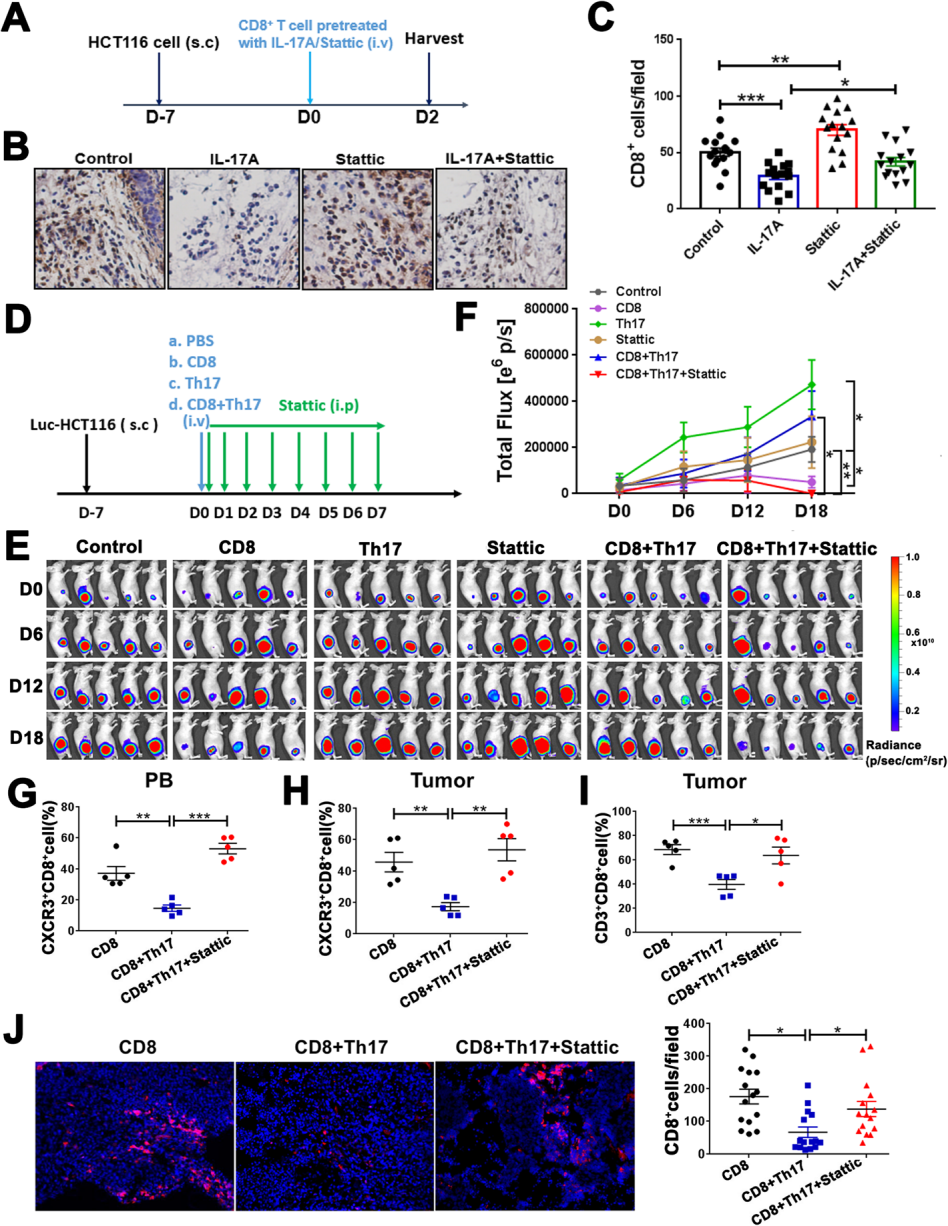
Blocking the STAT3 pathway rescues CD8^+^ T cell recruitment inhibited by Th17 cells in vivo. **a** Experimental scheme for the subcutaneous carcinoma model of BALB/c nude mice. CD8^+^ T cell were pretreated with rhIL-17A and/or Stattic for 48 h in vitro. Then the pretreated CD8^+^ T cells were intravenously injected on day 0 and tumors were harvested on day 2. Mice were divided into four groups (*n* = 5/group) based on CD8^+^ T cell pretreatment. **b** Immunohistochemistry of CD8 in tumor tissues; magnification, × 200. **c** CD8^+^ T cells were quantified using the ImagePro Plus software (Media Cybernetics) and expressed as the positive cells in 20 high-powered fields imaged by microscopy. Three fields were evaluated in one slice. **d** Experimental scheme for the subcutaneous carcinoma BALB/c nude mouse model. CD8^+^ T cells, Th17 cells, or CD8^+^ T cells + Th17 cells, were intravenously injected on day 0. Stattic was administrated intraperitoneally on day 0–7. Tumors were harvested on day 18. Mice were divided into six groups (*n* = 5/group). **e**, **f** Quantitative photon counting analysis of tumor progression by an in vivo imaging system. **g**–**i** CD8^+^ and CXCR3^+^CD8^+^ T cell expression in tumors and blood were examined by flow cytometry analysis. **j** Immunofluorescence of CD8 (red) expression in tumor tissues. Three fields were evaluated in one slice

To further examine the role of IL-17A/STAT3 signaling on CD8^+^ T cell migration in vivo, we utilized a xenograft mouse model of CRC. Tumor-bearing mice were intravenously injected with purified CD8^+^ T cells, which were pretreated with rhIL-17A or Stattic for 48 h in vitro (Fig. 6a). After 48 h, CD8^+^ T cell infiltration in tumor tissues was examined via immunohistochemistry. IL-17A suppressed the infiltration of CD8^+^ T cells in tumor tissues, which was attenuated by Stattic (Fig. 6b, c). To further examine whether blockade of STAT3 signaling can decrease tumor progression by rescuing the Th17-induced inhibition of CD8^+^ T cell infiltration in tumor tissues, Stattic was administered by intraperitoneal injection to Luc-GFP-HCT116-tumor bearing mice (Fig. 6d). CD8^+^ T cell infusion suppressed tumor growth; this effect was significantly diminished when CD8^+^ T cells were injected with Th17 cells (Fig. 6e, f). Consistently, tumor growth was significantly suppressed after Stattic was injected (Fig. 6e, f). Notably, we found that Th17 cells decreased CXCR3 expression levels on CD8^+^ T cells in PB, which was rescued by Stattic (Fig. 6g); CXCR3^+^CD8^+^ T cell infiltration in tumor tissues was consistent with these results (Fig. 6h). Moreover, flow cytometry and immunofluorescence indicated that CD8^+^ T cells were expressed at lower levels in the co-injection group of CD8^+^ T cells and Th17 cells and elevated in the Stattic group (Fig. 6i, j). These findings indicate that Th17 cells promote CRC tumor progression by partly blocking CXCR3^+^CD8^+^ T cell homing to tumor tissues via STAT3 signaling. Thus, STAT3 signaling may serve as a potential therapeutic target for CRC treatment.

### High expression levels of CD8 and CXCR3 and low IL-17A levels in the tumor stroma predict better OS of CRC patients

**Fig. 7:**
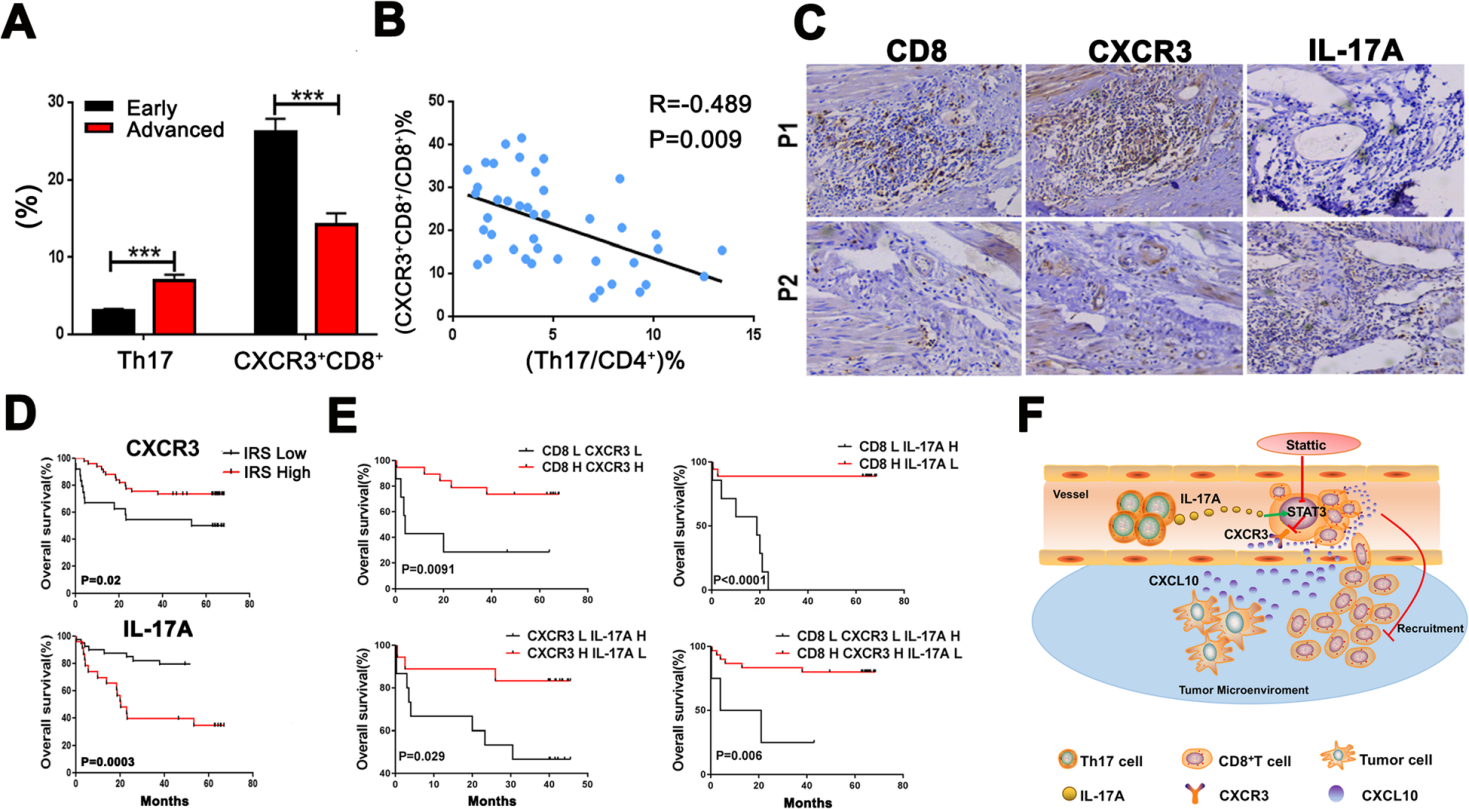
Increased expression of CD8 and CXCR3 and low IL-17A levels in the tumor stroma predicts better survival for CRC patients. **a** The expression levels of Th17 and CXCR3^+^CD8^+^ T cells in tumor tissues from early- and advanced-stage CRC patients were analyzed by flow cytometry. **b** Correlation between the concentration of the percentage of Th17 cells in CD4^+^T cells and CXCR3^+^CD8^+^ T cells in CD8^+^ T cells. Data were analyzed by Spearman’s rank correlation. **c** Expression of CD8, CXCR3, and IL-17A was measured in consecutive tumor sections of two patients by immunohistochemistry. **d** OS analysis of CRC patients with high and low IL-17A and CXCR3 expression (*n* = 70) presented as a Kaplan-Meier curve. High, IRS > 4; low, IRS ≤ 4. **e** OS of CRC patients with differential expression of CXCR3, CD8, and IL-17A. H, high, IRS > 4; L, low, IRS ≤ 4. **f** Diagram of the proposed mechanism. In the CRC circulation, Th17 cell-derived IL-17A activates the STAT3 pathway in CD8^+^ T cells, resulting in decreased CXCR3 expression and inhibiting migration of CD8^+^ T cells toward the CXCL10 secreted by tumor cells in tumor tissues. Targeting the STAT3 pathway leads to infiltration of CD8^+^ T cells into tumor tissues, eventually attenuating tumor progression

To investigate the clinical significance of Th17 cells and CXCR3^+^CD8^+^ cells in CRC patients, tumor tissues were collected. The results showed that expression levels of Th17 cells were significantly higher in advanced-stage than in early-stage tumor tissues, whereas the levels of CXCR3^+^CD8^+^ cells were lower (Fig. 7a). Previous studies have shown that CXCR3 is expressed on both tumor cells and stromal cells [43, 44]; therefore, we analyzed the distribution of CXCR3-positive cells in CRC tissues via immunofluorescence and found that cells highly expressing CXCR3 were predominantly located in stromal cells rather than in tumor cells (Supplementary Figure 6A, B). In the stroma, cells highly expressing CXCR3 were strongly co-localized with CD8, indicating that CXCR3 is highly expressed on CD8^+^ T cells and may be associated with the antitumor immune response (Supplementary Figure 6C). Given that IL-17A-positive cells were mainly derived from the CD4^+^ cell population, Th17 cells may be the primary source of IL-17A in tumor tissues (Supplementary Figure 6D). The expression levels of Th17 cells were negatively associated with the levels of CXCR3^+^CD8^+^ cells using correlation analysis (Fig. 7b). In the CRC tissue sections, a positive correlation between CXCR3 and CD8 was observed (Fig. 7c), while a negative correlation between IL-17A with CXCR3 and CD8 was identified (Fig. 7c). According to the IRS and positive rate, CRC patients with high levels of IL-17A in tumor tissues had a poor prognosis. In patients with higher CXCR3 expression, OS was significantly improved (Fig. 7d). According to the expression levels of CD8, CXCR3, and IL-17A, we divided all patients into different groups and performed survival analysis, which indicated that the following four groups have better prognosis: high CXCR3 and CD8 expression group, high CD8 and low IL-17A expression group, high CXCR3 and low IL-17A expression group, and high CD8 and CXCR3 and low IL-17A expression group (Fig. 7e). These findings further support the correlation between IL-17A, CXCR3, and CD8 in CRC tumor tissues.

Overall, our findings support a model wherein Th17 cells inhibit the expression of CXCR3 on CD8^+^ T cells in blood circulation by secreting IL-17A, which activates STAT3 signaling and downregulates CD8^+^ T cell infiltration in tumor tissues; these effects can be attenuated via Stattic (Fig. 7f).

## Discussion

As a common malignant tumor, the tumor-immune interactions of CRC have been intensely studied to achieve successful immunotherapeutic strategies. A number of studies have linked high T cell infiltration to improved survival [45–47]. Moreover, we found that CD8^+^ T cell density in tumor tissues was lower in advanced-stage CRC patients than in early-stage patients, which is in agreement with a previous study [5]. Thus, improving CD8^+^ T cell infiltration is a critical challenge for tumor immunotherapy.

CXCL10/CXCR3 signaling was found associated with paracrine interactions regulating leukocyte trafficking [44, 48]. However, CXCL10/CXCR3 expression during CRC progression was unknown. We found increased CXCL10 expression but decreased CD8^+^ T cell infiltration in tumor tissues of advanced-stage compared with early-stage CRC patients. The high levels of CXCL10 in tumor tissues are in line with a previous report that showed CXCL10 upregulation in metastatic tumors and demonstrated its functional role in tumor cell migration and invasion [49]. Interestingly, we found that CXCR3 expression on CD8^+^ T cells was significantly reduced in advanced-stage CRC patients, which was accompanied with decreased CD8^+^ T cell infiltration. We demonstrated the importance of CXCR3 expression for CD8^+^ T cells migration, which was consistent with previous studies that showed reduced homing of CD8^+^ T cells and other effector immune cells to tumors of CXCR3^−/−^ mice [15, 50]. Taken together, these findings indicate that decreased expression of CXCR3 on CD8^+^ T cells during tumor progression may play an important role in CD8^+^ T cell infiltration in patients with advanced-stage tumors.

Cancer-associated inflammation has been identified as a key determinant of disease progression and survival in CRC [51, 52]. We thus theorized that inflammatory factors may play a vital role in regulating CXCR3 expression on CD8^+^ T cells. In the IL-10^−/−^ colitis mouse model, CXCR3 was found highly expressed in the gut [53]. However, Singh et al. [54] reported that CXCR3^+^ T cells were reduced in IL-10^−/−^ chronic colitis mice, which led to the amelioration of chronic colitis. Moreover, CXCR3 expression on CD4^+^ T cells was decreased after treatment with IL-4 or IL-10 [40, 42]. IL-15 also activated CXCR3^+^ innate CD8^+^ T cells, which was associated with enhanced proliferation of CXCR3^+^ T cells [41]. However, few articles have reported the relationship between IL-17A and CXCR3 expression on CD8^+^ T cells. In this study, we found that CXCR3 expression on CD8^+^ T cells was inhibited by IL-17A, which further led to a decrease in CD8^+^ T cell infiltration.

CXCR3 expression is subject to transcriptional regulation driven by cues in the TME. Activation of multiple transcriptional factors, including STAT1, STAT3, and P65, drives CXCR3 expression on CD8^+^ T cells [55–57]. The mechanisms regulating CXCR3 expression, particularly expression induced by IL-17A, on CD8^+^ T cells in cancer remain largely unknown. Via multiplex screening assays, we found that IL-17A activated STAT3 in CD8^+^ T cells. Interestingly, a recent study reported that ablating STAT3 in CD8^+^ T cells prior to the CD8^+^ T cell transfer allows for efficient CD8^+^ T cell tumor infiltration and robust proliferation, resulting in increased tumor antigen-specific T cell activity and tumor growth inhibition [58]. Moreover, targeting the STAT3 gene in myeloid cells resulted in a reduction of Tregs and an increase in tumor-infiltrated CD8^+^ T cells, which led to effective antitumor immune responses [59, 60]. CD8^+^ T cells were also found accumulated in tumor sites of STAT3^−/−^ mice [57, 61]. This in agreement with our results where CXCR3 expression was rescued after treating IL-17A-incubated CD8^+^ T cells in vitro and in vivo with the STAT3 inhibitor Stattic, which also led to increased CD8^+^ T cell migration. Thus, the downregulated CXCR3 expression mediated by IL-17A is STAT3 dependent in CRC patients. However, it remains unknown whether STAT3 can induce or decrease CXCR3 expression on CD8^+^ T cells in a clinical setting.

Although we reported CD8^+^ T cell infiltration was systematically inhibited by Th17 cells via IL-17A/STAT3/CXCR3 axis, there are still several limitations to our study. To verify the role of IL-17A/STAT3 signaling on CD8^+^ T cell migration and tumor progression in vivo, we used immune-cell-infused xenograft mouse model according to previous studies [62, 63], which may not represent tumor immunity. A syngeneic model and specific tumor immunity should be extended to strengthen our hypothesis. Additionally, the technique for IL-17 secretion to enrich Th17 population [64–66] used in our study may not really represent the gamut of Th17 cells in human and limited quantities of a few other cytokines. Enhancing the efficiency of polarization of CD4^+^ memory T cells toward Th17 phenotype or using novel technique to sort Th17 cells should be adopted for further study.

## Conclusion

In this study, we reported a novel mechanism by which IL-17A inhibited the infiltration of CD8^+^ T cells by systematically downregulating CXCR3 expression on CD8^+^ T cells. IL-17A, predominantly secreted from Th17 cells, inhibited STAT3-dependent CXCR3 expression on CD8^+^ T cells, leading to decreased CD8^+^ T cell migration to tumor tissues. Thus, targeting STAT3 may offer exciting therapeutic opportunities for overcoming Th17-mediated immunosuppression. Our findings also provide potential strategies for converting cold tumors to hot tumors and improving immunotherapy.

## Supplementary information

**Additional file 1: Table S1**. Primers used in this study. **Figure S1**. The migratory ability of CD8^+^ T cells to CXCL10 depends on CXCR3 expression. **Figure S2**. The expression of CXCR3 ligands were unchanged after IL-17A stimulation. **Figure S3**. High expression levels of P-STAT3 in CD8^+^ T cells of advanced-stage CRC. **Figure S4**. IL-17A is predominantly secreted by CD4^+^ T cells from PB of CRC patients. **Figure S5**. The positive efficiency of enriched Th17 cells. **Figure S6**. CXCR3 is predominantly expressed in the stromal CD8^+^ T cells of tumor tissues, and IL-17A is mainly secreted by CD4^+^ T cells

## Data Availability

The datasets used and/or analyzed during the current study are available from the corresponding author on reasonable request.

## Abbreviations

CRC: Colorectal cancer
OS: Overall survival
TME: Tumor microenvironment
NK: Natural killer
PB: Peripheral blood
PBMCs: Peripheral blood mononuclear cells
HD: Healthy donors
ELISA: Enzyme linked immunosorbent assay
qRT-PCR: Quantitative reverse-transcription PCR
TF: Transcription factor
