# Supplementary material for: Th17 cells inhibit CD8+ T cell migration by systematically downregulating CXCR3 expression via IL-17A/STAT3 in advanced-stage colorectal cancer patients
Source: J Hematol Oncol. 2020 Jun 5;13:68. doi: 10.1186/s13045-020-00897-z (PMC7275425; doi:10.1186/s13045-020-00897-z)
Supplement: Supplementary file 1 — Additional file 1: Table S1. Primers used in this study. Figure S1. The migratory ability of CD8+ T cells to CXCL10 depends on CXCR3 expression. Figure S2. The expression of CXCR3 ligands were unchanged after IL-17A stimulation. Figure S3. High expression levels of P-STAT3 in CD8+ T cells of advanced-stage CRC. Figure S4. IL-17A is predominantly secreted by CD4+ T cells from PB of CRC patients. Figure S5. The positive efficiency of enriched Th17 cells. Figure S6. CXCR3 is predominantly expressed in the stromal CD8+ T cells of tumor tissues, and IL-17A is mainly secreted by CD4+ T cells [file 13045_2020_897_MOESM1_ESM.zip › Supplementary material.docx]

**Table S1: Primers used in this study**

| **Gene name** | **Forward primer** | **Reverse primer** | **Product length** |
| --- | --- | --- | --- |
| GAPDH | GCACCGTCAAGGCTGAGAAC | TGGTGAAGACGCCAGTGGA | 138 bp |
| CCL4 | CTGTGCTGATCCCAGTGAATC | TCAGTTCAGTTCCAGGTCATACA | 61 bp |
| CCL5 | CCAGCAGTCGTCTTTGTCAC | CTCTGGGTTGGCACACACTT | 54 bp |
| CCL8 | TGGAGAGCTACACAAGAATCACC | TGGTCCAGATGCTTCATGGAA | 133 bp |
| CCL11 | CCCCTTCAGCGACTAGAGAG | TCTTGGGGTCGGCACAGAT | 109 bp |
| CCL17 | AGCCATTCCCCTTAGAAAGC | CTGCCCTGCACAGTTACAAA | 90 bp |
| CCL21 | GTTGCCTCAAGTACAGCCAAA | AGAACAGGATAGCTGGGATGG | 102 bp |
| CXCL9 | CCAGTAGTGAGAAAGGGTCGC | AGGGCTTGGGGCAAATTGTT | 99 bp |
| CXCL10 | GTGGCATTCAAGGAGTACCTC | TGATGGCCTTCGATTCTGGATT | 198 bp |
| CXCL11 | CCTTGGCTGTGATATTGTGTGC | CCTATGCAAAGACAGCGTCCT | 148 bp |

**Supplementary Materials and Methods**

**Mononuclear cells isolation from tumors**

Tumor samples were dissociated into single-cell suspensions, using the Tumor Dissociation Kit, human and the gentle MACS Octo Dissociator with Heaters. Tissue samples were cut into 1mm^3^ pieces and 2.5 mL digest solution were used per sample. All other steps were carried out according to the protocol included in the kit (130-095-929; Miltenyi Biotec). Mononuclear cell isolation is commonly done via density gradient centrifugation over Ficoll-Hypaque.

**Supplementary Figure legends**

**Figure S1. The migratory ability of CD8^+^ T cells to CXCL10 depends on CXCR3 expression.**

CXCR3^+^CD8^+^ and CXCR3^-^CD8^+^ cells were sorted from PBMC of HDs. The migratory ability of CD8^+^ T cells with different ratios of CXCR3^+^CD8^+^ to CXCR3^-^CD8^+^ cells (1:1, 1:2, 1:4 and 1:8) was analyzed in the presence of different concentrations of rhCXCL10 (10 ng/ml, 20 ng/ml and 50ng/ml). *P < 0.05, **P < 0.001, ***P < 0.0001.

**Figure S2. The expression of CXCR3 ligands were unchanged after IL-17A stimulation.** Relative expression levels of CXCL9、CXCL10 and CXCL11 after IL-17A ( 20 ng/mL ) treatment for 24h were determined by real-time PCR. NS, non-significant.

**Figure S3. High expression levels of P-STAT3 in CD8^+^ T cells of advanced-stage CRC.**

(A, B) The percentage of P-STAT3^+^CD8^+^ T cells to total CD8^+^ T cells from PB of CRC patients and HDs (A) as well as early-stage and advanced-stage CRC patients (B). *P < 0.05, ***P < 0.0001.

**Figure S4.** **IL-17A is predominantly secreted by CD4^+^ T cells from PB of CRC patients.**

(A, B) Representative images of PBMCs stained for CD4 (red), CD8 (red), IL-17A (green), and DAPI (blue). Closed arrows represent double positive T cells. C. The percentage of IL-17A positive cells in CD4^+^ and CD8^+^ T cells were analyzed.

**Figure S5.** **The positive efficiency of enriched Th17 cells.**

(A, B) The Th17 cells were induced and enriched from CD4^+^ memory T cells. Flow cytometry analysis of IL-17A-secreting CD4^+^ T cells before and after treatment. ***P < 0.0001

**Figure S6. CXCR3 is predominantly expressed in the stromal CD8^+^ T cells of tumor tissues, and IL-17A is mainly secreted by CD4^+^ T cells.**

(A) Representative images of CXCR3 (red) expression in tumor tissues by immunofluorescence. (B) The percentage of CXCR3 positive cells in stromal and tumor cells were analyzed. (C, D) Representative images of tumor tissues stained for CXCR3, CD4 (red), CD8, IL-17A (green), and DAPI (blue). ***P < 0.0001.
