# Supplementary figures and images for: Th17 cells inhibit CD8+ T cell migration by systematically downregulating CXCR3 expression via IL-17A/STAT3 in advanced-stage colorectal cancer patients
Source: J Hematol Oncol. 2020 Jun 5;13:68. doi: 10.1186/s13045-020-00897-z (PMC7275425; doi:10.1186/s13045-020-00897-z)

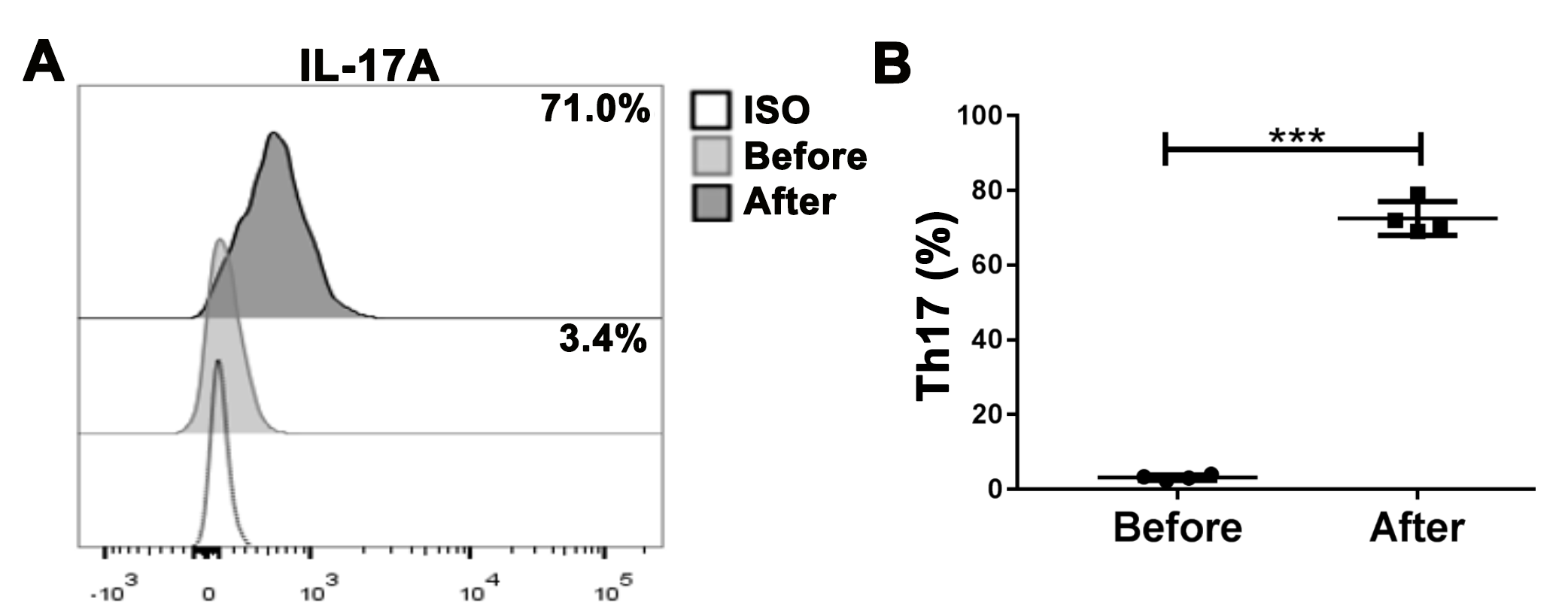

Supplement: Supplementary file 1 — Additional file 1: Table S1. Primers used in this study. Figure S1. The migratory ability of CD8+ T cells to CXCL10 depends on CXCR3 expression. Figure S2. The expression of CXCR3 ligands were unchanged after IL-17A stimulation. Figure S3. High expression levels of P-STAT3 in CD8+ T cells of advanced-stage CRC. Figure S4. IL-17A is predominantly secreted by CD4+ T cells from PB of CRC patients. Figure S5. The positive efficiency of enriched Th17 cells. Figure S6. CXCR3 is predominantly expressed in the stromal CD8+ T cells of tumor tissues, and IL-17A is mainly secreted by CD4+ T cells [file 13045_2020_897_MOESM1_ESM.zip › S FIG5.tif]

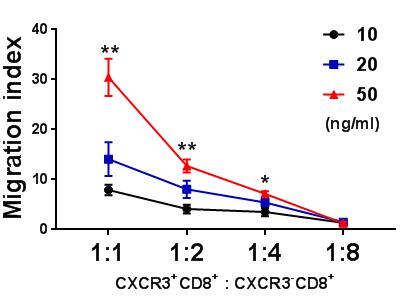

Supplement: Supplementary file 1 — Additional file 1: Table S1. Primers used in this study. Figure S1. The migratory ability of CD8+ T cells to CXCL10 depends on CXCR3 expression. Figure S2. The expression of CXCR3 ligands were unchanged after IL-17A stimulation. Figure S3. High expression levels of P-STAT3 in CD8+ T cells of advanced-stage CRC. Figure S4. IL-17A is predominantly secreted by CD4+ T cells from PB of CRC patients. Figure S5. The positive efficiency of enriched Th17 cells. Figure S6. CXCR3 is predominantly expressed in the stromal CD8+ T cells of tumor tissues, and IL-17A is mainly secreted by CD4+ T cells [file 13045_2020_897_MOESM1_ESM.zip › S FIG1.jpg]

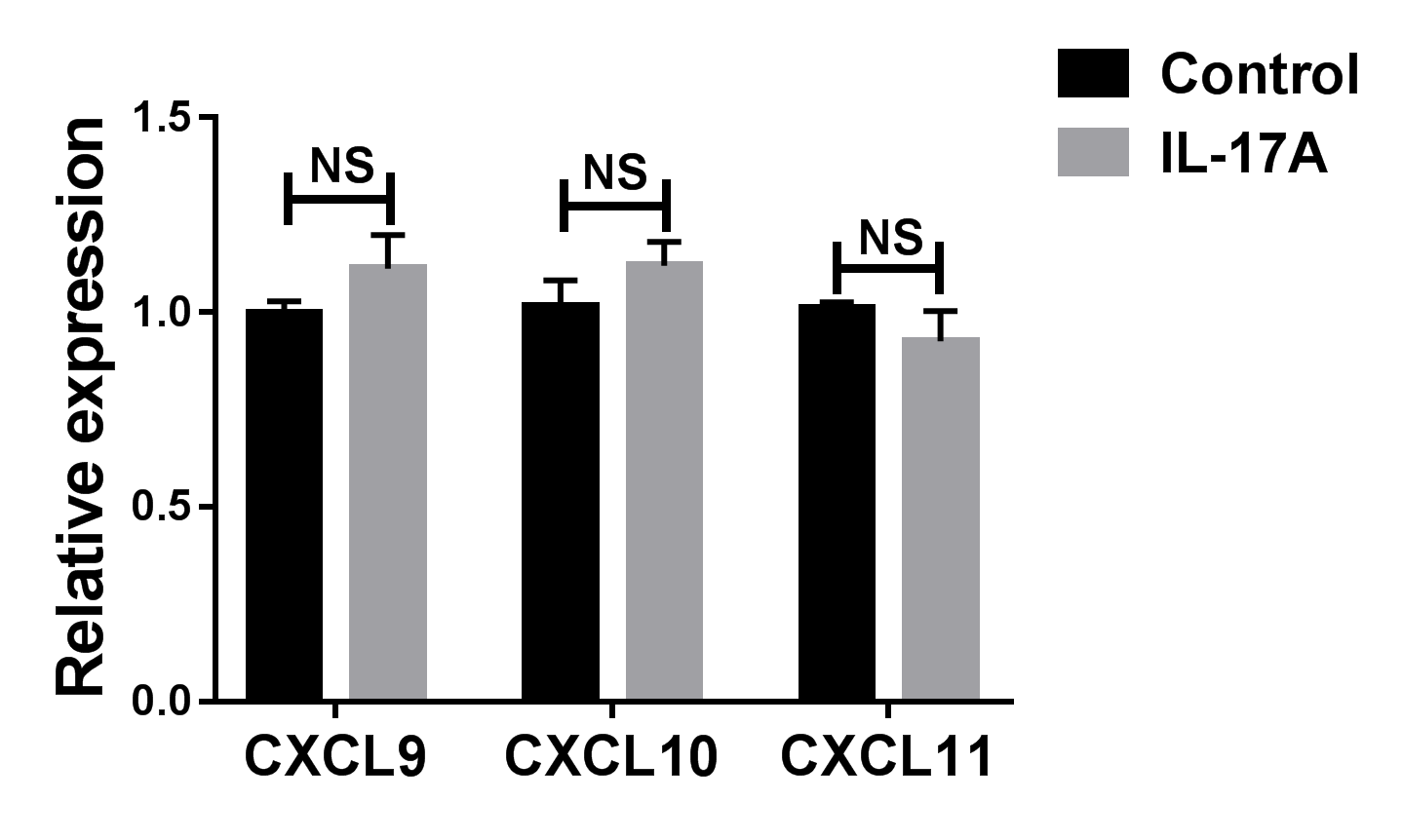

Supplement: Supplementary file 1 — Additional file 1: Table S1. Primers used in this study. Figure S1. The migratory ability of CD8+ T cells to CXCL10 depends on CXCR3 expression. Figure S2. The expression of CXCR3 ligands were unchanged after IL-17A stimulation. Figure S3. High expression levels of P-STAT3 in CD8+ T cells of advanced-stage CRC. Figure S4. IL-17A is predominantly secreted by CD4+ T cells from PB of CRC patients. Figure S5. The positive efficiency of enriched Th17 cells. Figure S6. CXCR3 is predominantly expressed in the stromal CD8+ T cells of tumor tissues, and IL-17A is mainly secreted by CD4+ T cells [file 13045_2020_897_MOESM1_ESM.zip › s FIG2.tif]

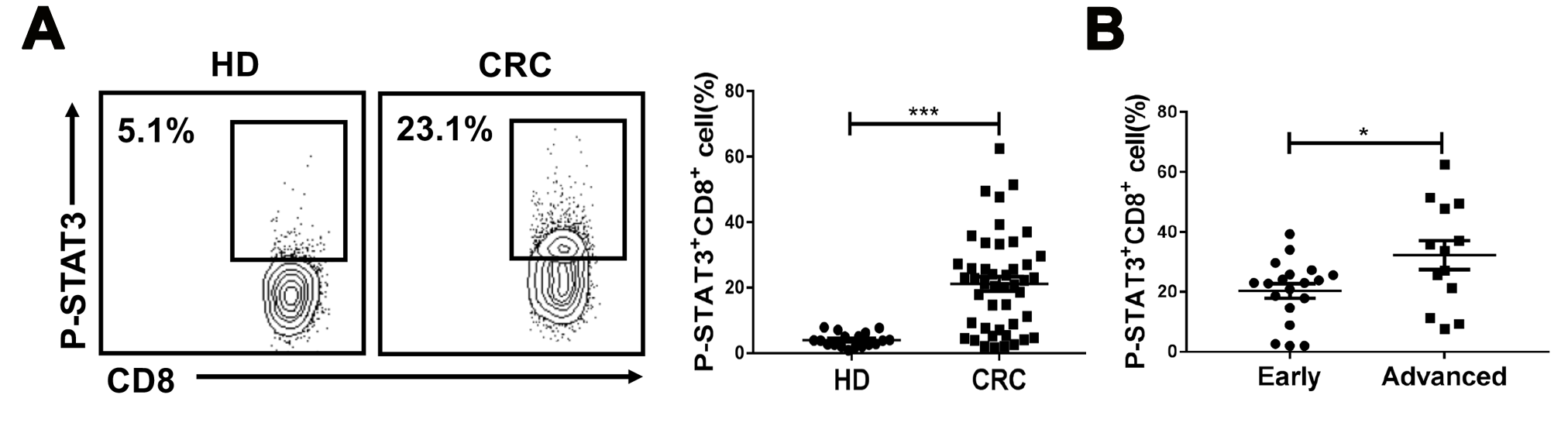

Supplement: Supplementary file 1 — Additional file 1: Table S1. Primers used in this study. Figure S1. The migratory ability of CD8+ T cells to CXCL10 depends on CXCR3 expression. Figure S2. The expression of CXCR3 ligands were unchanged after IL-17A stimulation. Figure S3. High expression levels of P-STAT3 in CD8+ T cells of advanced-stage CRC. Figure S4. IL-17A is predominantly secreted by CD4+ T cells from PB of CRC patients. Figure S5. The positive efficiency of enriched Th17 cells. Figure S6. CXCR3 is predominantly expressed in the stromal CD8+ T cells of tumor tissues, and IL-17A is mainly secreted by CD4+ T cells [file 13045_2020_897_MOESM1_ESM.zip › S FIG3.tif]

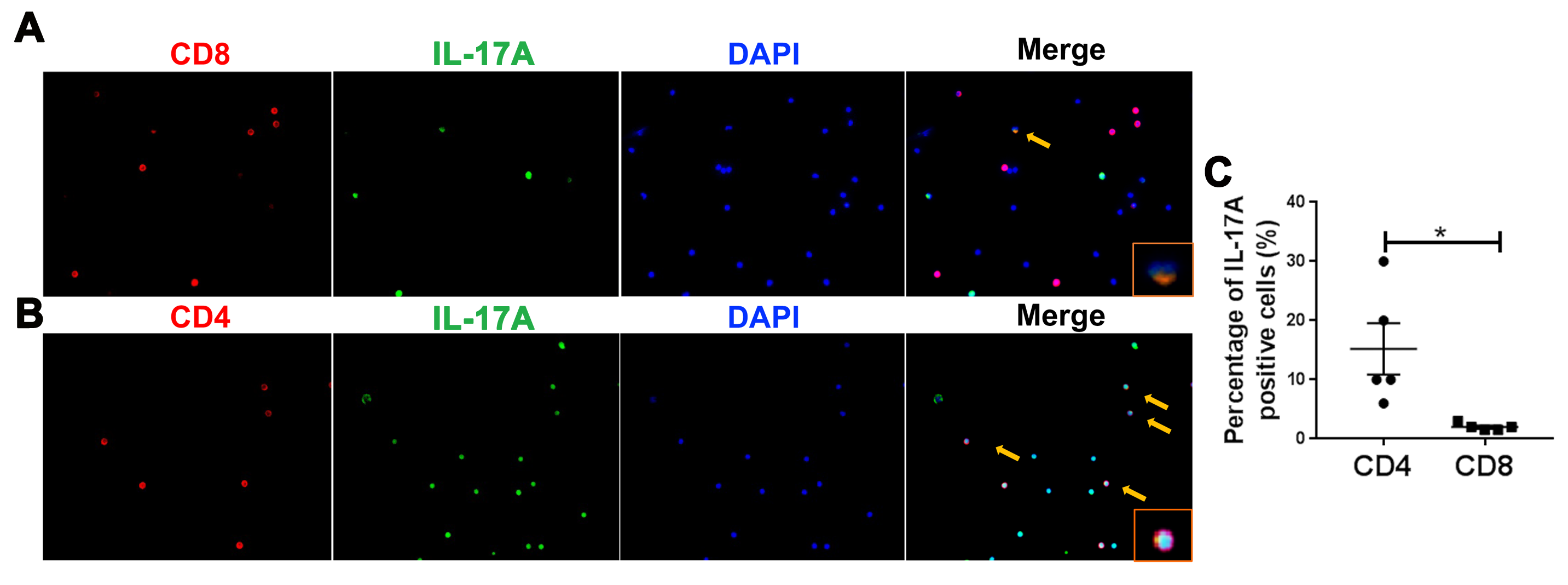

Supplement: Supplementary file 1 — Additional file 1: Table S1. Primers used in this study. Figure S1. The migratory ability of CD8+ T cells to CXCL10 depends on CXCR3 expression. Figure S2. The expression of CXCR3 ligands were unchanged after IL-17A stimulation. Figure S3. High expression levels of P-STAT3 in CD8+ T cells of advanced-stage CRC. Figure S4. IL-17A is predominantly secreted by CD4+ T cells from PB of CRC patients. Figure S5. The positive efficiency of enriched Th17 cells. Figure S6. CXCR3 is predominantly expressed in the stromal CD8+ T cells of tumor tissues, and IL-17A is mainly secreted by CD4+ T cells [file 13045_2020_897_MOESM1_ESM.zip › S FIG4.tif]

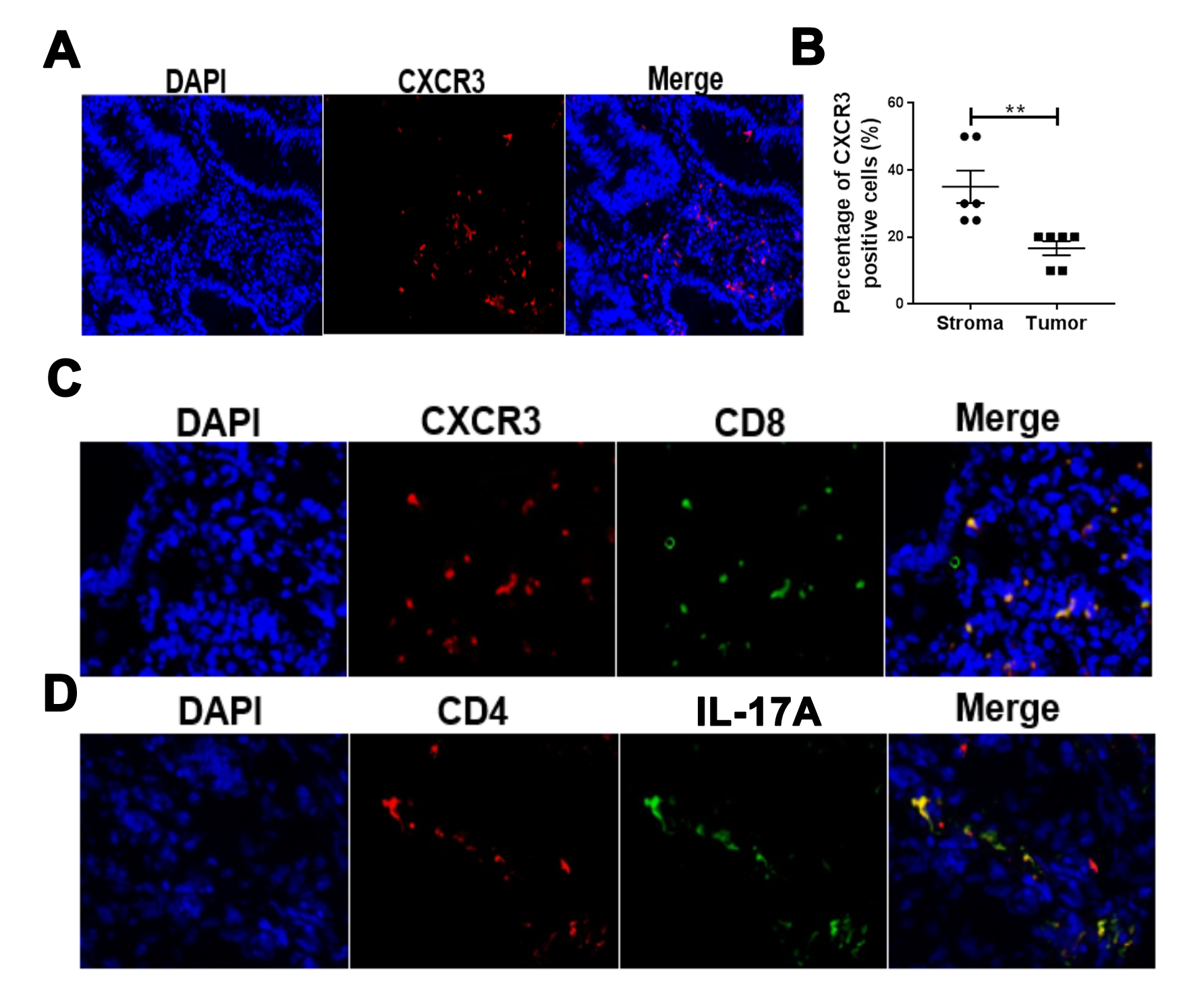

Supplement: Supplementary file 1 — Additional file 1: Table S1. Primers used in this study. Figure S1. The migratory ability of CD8+ T cells to CXCL10 depends on CXCR3 expression. Figure S2. The expression of CXCR3 ligands were unchanged after IL-17A stimulation. Figure S3. High expression levels of P-STAT3 in CD8+ T cells of advanced-stage CRC. Figure S4. IL-17A is predominantly secreted by CD4+ T cells from PB of CRC patients. Figure S5. The positive efficiency of enriched Th17 cells. Figure S6. CXCR3 is predominantly expressed in the stromal CD8+ T cells of tumor tissues, and IL-17A is mainly secreted by CD4+ T cells [file 13045_2020_897_MOESM1_ESM.zip › S FIG6.tif]
